# Activation of Group II Metabotropic Glutamate Receptors Suppresses Excitability of Mouse Main Olfactory Bulb External Tufted and Mitral Cells

**DOI:** 10.3389/fncel.2017.00436

**Published:** 2018-01-17

**Authors:** Hong-Wei Dong, Matthew Ennis

**Affiliations:** Department of Anatomy and Neurobiology, University of Tennessee Health Science Center, Memphis, TN, United States

**Keywords:** mGluRs, mGluR2, external tufted cell, mitral cell, glomerulus, mouse main olfactory bulb

## Abstract

Metabotropic glutamate receptors (mGluRs) are abundantly expressed in the rodent main olfactory bulb. The function of Group I mGluRs has been investigated in a number of studies, while the actions of Group II mGluRs, which include the mGluR2 and mGluR3 subtypes, have been less well explored. Here, we used electrophysiological approaches in mouse olfactory bulb slices to investigate how Group II mGluR activation and inactivation modifies the activity of external tufted (ET) and mitral cells. The Group II mGluR agonist DCG-IV was found to directly and uniformly reduce the spontaneous discharge of ET and mitral cells. The inhibitory effect of DCG-IV was absent in mitral cells with truncated apical dendrites, indicating a glomerular site of action. DCG-IV did not influence olfactory nerve-evoked monosynaptic responses in ET or mitral cells, indicating that Group II mGluRs do not presynaptically modulate glutamate release from olfactory nerve terminals. In contrast, DCG-IV suppressed polysynaptic responses in periglomerular cells evoked by olfactory nerve stimulation. DCG-IV also inhibited glutamate release from ET cells, and suppressed the spontaneous and olfactory nerve-evoked long-lasting depolarization in mitral cells. Applied alone, Group II receptor antagonists were without effect, suggesting that basal activation of these receptors is nil. These findings suggest that Group II mGluRs inhibit ET and mitral cell activity and further dampen intraglomerular excitatory circuits by suppressing glutamate release.

## Introduction

Neurons in main olfactory bulb (MOB) express members of the three-metabotropic glutamate receptor (mGluR) family groups designated as: Group I (mGluR1 and 5), Group II (mGluR2 and 3) and Group III (mGluR4 and 6-8) (Conn and Pin, 1997). The function of Group I mGluRs (mGluR1 and 5) in the MOB has been explored in a number of studies and has been linked to excitation of mitral/tufted and granule cells, slow oscillatory activity in response to olfactory nerve input and prolonged odor responses (Schoppa and Westbrook, 2001; Heinbockel et al., 2004, 2007; Yuan and Knopfel, 2006; Dong et al., 2007, 2009; Matsumoto et al., 2009).

Group II mGluRs in the MOB have been less explored. Anatomically, mGluR3 mRNA expression is negligible in the MOB, with only few scattered neurons of unknown identity observed (Ohishi et al., 1993). mGluR2 protein or mRNA localization studies report the presence of labeled neuron in all MOB layers, with substantial populations in the glomerular, mitral and granule cell layers (Ohishi et al., 1993, 1998). Labeled neurons were not definitively identified however, and the intense neuropil staining with mGluR2 immunocytochemistry in these and other studies (Neki et al., 1996) made cellular expression difficult. Studies using nonspecific mGluR2/3 antibodies (Petralia et al., 1996; Sahara et al., 2001) yielded results comparable to mGluR2 specific antibodies, suggesting that mGluR2 is the only Group II receptor with a significant functional role in the MOB.

Although Group II mGluRs play a well characterized disinhibitory role in the accessory olfactory bulb where they suppress GABA release at granule-to-mitral cell dendrodendritic synapses (Hayashi et al., 1993), relatively little is known about their function in the MOB. A recent study indicates mGluR2 acts similarly in the MOB to suppress GABA release from granule and periglomerular (PG) cells (Zak et al., 2015). The impact of Group II mGluRs on other elements of the MOB circuitry remain unexplored. The present study therefore investigated the actions of Group II mGluR activation and inactivation on external tufted (ET) and mitral cells using electrophysiological approaches in mouse MOB slices. Our findings indicate that Group II mGluR activation suppresses the excitability of both cell types.

## Materials and methods

### Slice preparation

Male and female 21–42 day old C57BL/6J mice were obtained from Jackson laboratory. All animals were housed in a vivarium that was maintained between 22 and 23°C and under a 12-h light on/off cycle. Food and water were available *ad libitum*. All experimental procedures described below were reviewed and approved by Institutional Animal Care and Use Committee of University of Tennessee Health Science Center in accordance with and National Institutes of Health guidelines. Mice were decapitated and horizontal 400 μm-thick olfactory bulb slices were prepared as previously described (Dong and Ennis, 2014). Briefly, the olfactory bulbs were removed and immersed in oxygenated chilled sucrose-artificial cerebrospinal fluid (ACSF) composed of the following (in mM): 62 NaCl, 26 NaHCO_3_, 1.25 NaH_2_PO_4_, 3 KCl, 4 MgSO_4_, 0.1 CaCl_2_, 15 glucose, and 120 sucrose; pH 7.3, 310 mOsm. Horizontal slices were cut with a vibrating microtome (Leica VT 1200S, Leica, Germany) at thickness of 400 μm. After recovery at 33°C for 20 min, slices were incubated until used at room temperature (22°C) in normal ACSF equilibrated with carbogen (95% O_2_-5% CO_2_) and composed of the following (in mm): 126 NaCl, 26 NaHCO_3_, 3 KCl, 1.25 NaH_2_PO_4_, 2 MgCl_2_, 2 CaCl_2_, and 15 glucose (pH 7.3, 310 mOsm). For recording, a single slice was placed in a recording chamber and continuously perfused with carbogen-saturated ACSF at the rate of 1.5–2 ml/min. On average, it took ~1.5 min for drugs to arrive at the recording chamber and ~3 min to achieve stable effects.

### Electrophysiology

Recordings were performed at 30°C. Neurons were visualized using an upright microscope (BX50 WI; Olympus, Tokyo, Japan) equipped with epifluorescence and near-infrared differential interference contrast optics. Patch pipettes were fabricated from borosilicate glass and were pulled to a resistance of 3–5 MΩ for mitral cells, 5–7 MΩ for ET cells, and 7–9 MΩ for PG cells. Only neurons with access resistance less than 25 MΩ were included in this study. Unless otherwise noted, the intracellular solution for both voltage and current-clamp recordings was composed of (in mM): 124 potassium gluconate, 1 NaCl, 10 phosphocreatine di(tris) salt, 3 MgATP, 0.3 Na_2_GTP, 0.5 EGTA, and 10 HEPES; pH 7.3, 290 mOsm. The internal solution for self-excitation experiments was (in mM): 125 cesium methanesulfonate (CsMeSO_3_), 1 NaCl, 10 phosphocreatine di(tris) salt, 3 MgATP, 0.3 Na_2_GTP, 0.5 EGTA, 10 HEPES; pH 7.3, 290 mOsm. Extracellular recordings were obtained using patch pipettes filled with ACSF.

Cells recorded in whole cell mode were filled with Lucifer yellow (0.02%) for in situ determination of location and morphology. Recorded cells were identified by location, morphology and electrophysiological properties (Hayar et al., 2004a,b, 2005; Hayar and Ennis, 2007; Liu and Shipley, 2008a,b; Dong et al., 2009; Dong and Ennis, 2014). Briefly, ET cells were distinguished by (1) a relatively large soma and a primary dendrite with a tuft-like arborization that ramifies within a single glomerulus, (2) lack of secondary dendrites, and (3) distinct spontaneous rhythmic bursting. PG cells were identified by: (1) relatively small soma and a restricted dendritic arbor, (2) relatively high input impedance (~1,000 MΩ) compared to ET or mitral cells, and (3) spontaneous bursts of EPSPs or EPSCs. Mitral cells were identified by large soma in the mitral cell layer and an apical dendrite that arborized in a single glomerulus.

Whole cell voltage- or current-clamp recordings were made with a MultipleClamp 700B amplifier (Molecular Devices, Sunnyvale, CA). The junction potential was 9–10 mV, and all reported voltage measurements were uncorrected for these potentials. No series resistance compensation was performed. Analog signals were low-pass filtered at 2 kHz (MultiClamp 700B) and digitized at 5 kHz using a Digidata 1,440 A interface and pClamp 10.3 software (Molecular Devices). Miniature EPSCs (mEPSCs) were isolated by bath application of (in μM): 10 gabazine, 50 APV, 1 TTX. Robust self-excitation was evoked in ET cells by a brief depolarizing step (−60 to 0 mV, 5 ms) in normal ACSF with glutamate (10 μM) in the internal solution to avoid transmitter depletion (Ma and Lowe, 2007; De Saint Jan et al., 2009). Olfactory nerve (ON) stimulation (0.1 Hz, 100 μs in duration, 15–40 μA intensity) was via a bipolar concentric stainless steel electrode (25 μm in diameter) placed in the ON layer. Isolated constant current pulses from an ISO-Flex isolator (AMPI) were controlled by a Master-8 stimulator. Spontaneous EPSCs (sEPSCs) were recorded at a holding potential of −60 mV. EPSC bursts in PG cells were defined as a series of four or more consecutive sEPSC occurring at less than 30 ms intervals. A spike burst in ET cells was defined as a series of two or more consecutive spikes occurring at < 75 ms intervals (Dong et al., 2009). Detection of EPSCs, spikes and LLDs was performed off-line using Mini Analysis program (Synaptosoft, Decature, GA). All detected events were visually confirmed. To measure steady-state membrane potential during spontaneous spiking, traces were low pass filtered at 5 Hz and the membrane potential was averaged in a 30 s epoch. Data were expressed as mean ± SEM and statistically analyzed with paired or unpaired *t*-tests unless noted otherwise.

### Drugs and solutions

Unless otherwise noted, drugs and solutions were applied to the perfusion solution with a three-way valve system. Recording media, gabazine, 6-cyano-7-nitroquinoxaline-2,3-dione (CNQX), (±) 2-amino-5-phosphonopentanoic acid (APV) were obtained from Sigma-Aldrich (St. Louis, MO). 3, 4-dihydroxyphenylglycine (DHPG), 2′, 3′-dicarboxycyclopropyl glycine (DCG-IV), LY341495, tetrodotoxin (TTX) were purchased from Tocris Bioscience (Ellisville, MO).

## Results

### Activation of group II mGluRs suppresses ET cell bursting

**Figure 1:**
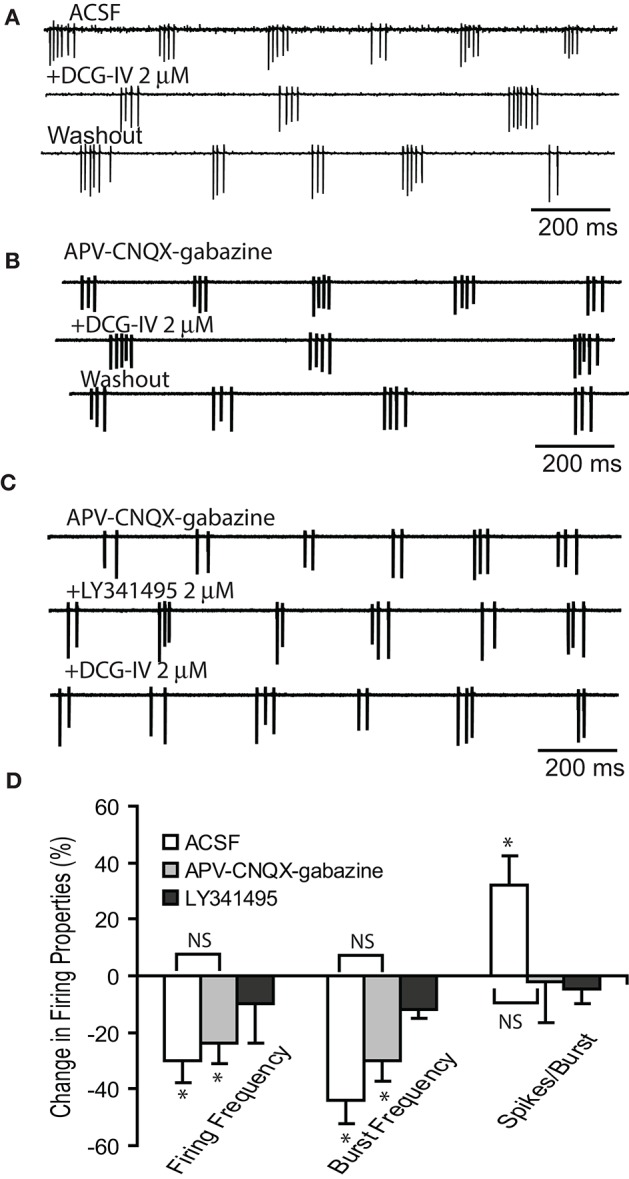
DCG-IV decreases ET cell bursting. **(A–C)** Extracellular recordings from example ET cells show that DCG-IV (2 μM) reversibly reduced the firing frequency and burst rate in in normal ACSF **(A)** and APV-CNQX-gabazine **(B)**, but had no effect when applied in the presence of LY341495 **(C)**. **(D)** Group data summarizing the effects of DCG-IV in the conditions in **(A–C)**; ACSF (*n* = 6), APV-CNQX-gabazine (*n* = 6) and LY341495+APV-CNQX-gabazine (*n* = 4). Data are expressed as percent change from respective control values. ^*^p < 0.05 vs. respective control, paired *t*-tests. NS, non-significant, Kruskal-Willis Test followed by Chi-Square tests; *p* = 0.81 for firing frequency, *p* = 0.47 for burst frequency, and *p* = 0.12 for number of spikes/burst.

Previous studies reported that activation of group I mGluRs with DHPG or the mixed group I/II/III mGluR agonist L-CCG-I directly increased ET cell rhythmic bursting (Ishida et al., 1993; Tomita et al., 2000; Kirschstein et al., 2004; Dong et al., 2009). Here, we examined if selective activation of group II mGluRs with the agonist DCG-IV (Salin et al., 2001; Taniguchi et al., 2013; Zak et al., 2015) modulated ET cell spontaneous bursting. Extracellular recordings were used to obviate burst rundown in ET cells that can occur during whole cell recordings. Bath applied DCG-IV (2 μM) significantly dampened ET cell firing properties (*n* = 6, Figures 1A,D), decreasing burst frequency by 44.3 ± 8.3% (2.6 ± 0.5 Hz to 1.6 ± 0.4 Hz, *p* < 0.01, paired *t*-test) and the mean firing frequency by 29.6 ± 7.6% (8.5 ± 2.0 to 6.4 ± 1.8 Hz, *p* < 0.05), while increasing number of spikes/burst by 32.4 ± 10.6% (2.9 ±0.3 to 4.0 ± 0.6, *p* < 0.05). Similar effects of DCG-IV were observed in the presence of CNQX (10 μM), APV (50 μM) and gabazine (10 μM), *n* = 6 (Figures 1B,D). Burst frequency decreased by 30.5 ± 7.3% (2.5 ± 0.6 Hz vs. 1.9 ± 0.7 Hz, *p* < 0.05 paired *t*-test), firing frequency decreased by 24.1 ± 6.0% (8.0 ± 1.8 Hz vs. 6.3 ± 1.7 Hz, *p* < 0.05 paired *t*-test); the number spikes/burst were unaffected (3.3 ± 0.3 vs. 3.2 ± 0.4, *p* > 0.05). In the presence of CNQX-APV-gabazine and LY341495 (2 μM), a selective Group II mGluR antagonist at low micromolar concentrations (Kingston et al., 1998), DCG-IV did not alter firing rate, burst rate or the number of spikes/burst (Figure 1C, D *p* > 0.05, *n* = 4, paired *t*-tests). These results show that activation of Group II mGluRs suppresses ET cell firing and this persists when ionotropic glutamate and GABA receptors are blocked (Figure 1D, *p* > 0.05 for all firing parameters, Kruskal-Willis Test followed by Chi-Square *post-hoc* tests). The latter observation suggests that the suppression of firing is due primarily to a direct action on ET cells. This result was also verified in current clamp recordings, where DCG-IV (2 μM) hyperpolarized ET cell membrane potential by −2.4 ± 0.6 mV (*p* < 0.05, paired *t*-test, *n* = 5, data not shown).

**Figure 2:**
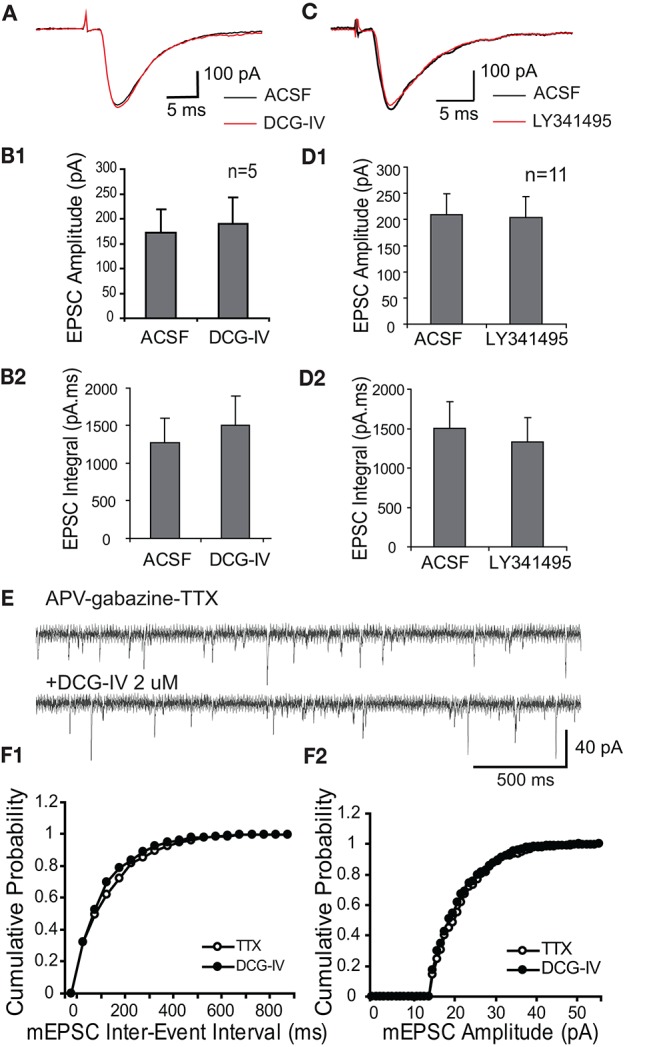
Group II mGluRs do not modulate ON-evoked or spontaneous EPSCs in ET cells. **(A)** The ON-evoked monosynaptic EPSC in an example ET cell (V_hold_ = −60 mV) is not altered by DCG-IV (2 μM); evoked responses in this and subsequent figures are averages of 10 traces unless otherwise noted. **(B)** Group data (*n* = 5) show that DCG-IV does not alter ON-evoked EPSC amplitude **(B1)** or integral **(B2)**. **(C)** As in **(A)**, the ON-evoked EPSC in an ET cell is not influenced by LY341495 (2 μM). **(D1,2)** Group data (*n* = 11) as in **(B)** for LY341495 experiments. **(E)** mEPSCS recorded from an ET cell (V_hold_ = −60 mV) in the presence of APV-gabazine-TTX were not affected by DCG-IV (2 μM). **(F1,2)** Cumulative inter-event **(F1)** and amplitude **(F2)** probability histograms show that DCG-IV did not affect mEPSC frequency or amplitude.

In order to assess the specificity of mGluR actions, we investigated if Group II mGluRs presynaptically influence glutamate release from ON terminals. We first examined if DCG-IV influences monosynaptic responses of ET cells to ON input (30–40 μA) in voltage clamp. ON stimulation evoked short and constant latency EPSCs in ET cells (1.9 ± 0.1 ms, *n* = 6, Figure 2A), consistent with monosynaptic mediation (Hayar et al., 2004b; Liu and Shipley, 2008b). DCG-IV (2 μM) did not significantly influence the peak amplitude or the integral of ON-evoked EPSCs (*n* = 5, Figures 2A,B): peak amplitude, 155.0 ± 51.7 pA vs. 178.0 ± 60.2 pA, *p* = 0.24, paired *t*-test); integral, 1,274 ± 321 pA.ms vs. 1,505 ± 389 pA.ms, *p* = 0.30, paired *t*-test). Similarly, LY341495 (2 μM), *n* = 11, Figures 2C,D) alone did not alter the ON-evoked EPSC peak amplitude (189.2 ± 39.3 vs. 190.7 ± 42.6 pA, *p* = 0.90, paired *t*-test) or integral (1,238.3 ± 232.8 pA.ms vs. 1,066.7 ± 181.4 pA.ms, *p* = 0.06, paired *t*-test).

Consistent with the lack of modulation of the ON-evoked EPSC, DCG-IV did not alter miniature EPSCs (mEPSCs) recorded in ET cells in the presence of APV (50 μM), gabazine (10 μM), and TTX (1 μM) (Figures 2E,F, *n* = 5). mEPSC amplitude (24.6 ± 4.6 pA vs. 24.3 ± 4.1 pA, *p* = 0.48), inter-event intervals (14.4 ± 4.1 ms vs. 14.5 ± 5.1 ms, *p* = 0.93), rise time (1.9 ± 0.1 ms vs. 2.0 ± 0.1 ms, *p* = 0.35) and decay time (6.2 ± 0.1 ms vs. 6.5 ± 0.2 ms, *p* = 0.39) were unaffected. Taken together, these results indicate that activation of Group II mGluRs suppresses ET cell firing, but does not presynaptically modulate glutamate release from ON terminals.

### Group I and II mGluRs differentially modulate mitral cell excitability

**Figure 3:**
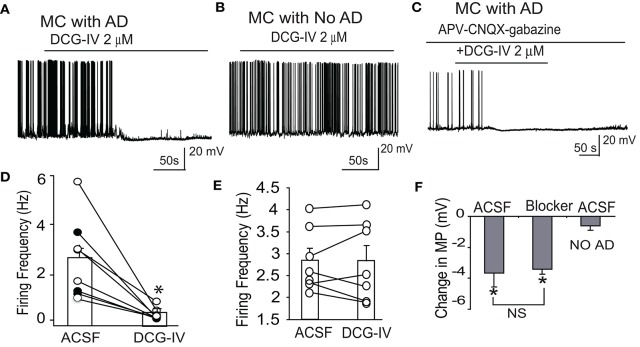
DCG-IV inhibits firing in mitral cells with intact apical dendrites. **(A)** Example current clamp record showing that 2 μM DCG-IV in normal ACSF terminates firing in a mitral cell (MC) with an intact apical dendrite (AD). **(B)** Firing in an example mitral cell with truncated apical dendrites is unaffected by DGG-IV. **(C)** DCG-IV applied in the presence of APV-CNQX-gabazine inhibited firing of this mitral cell with intact apical dendrites. **(D)** Group data showing the effects of DCG-IV on firing frequency of 8 mitral cells with intact apical dendrites. Bars show mean values; closed circles depict the 3 cells that stopped firing after DCG-IV. ^*^p < 0.05 vs. control, paired *t*-tests. **(E)** Recording from 7 MCs with apical dendrites truncated showing that DCG-IV had no effect on MC firing frequency. **(F)** Group data showing the effects of DCG-IV on membrane potential (MP) in mitral cells with intact dendrites in normal ACSF (*n* = 5) or APV-CNQX-gabazine (Blocker, *n* = 7), and in mitral cells with truncated apical dendrites in normal ACSF (No AD, *n* = 7). ^*^p < 0.05 vs. control, paired *t*-tests; NS, non-significant, Mann-Whitney *U*-test.

We next investigated the effects of DCG-IV on mitral cell activity. DCG-IV (2 μM) hyperpolarized mitral cells by −3.2 ± 1.0 mV (*p* < 0.05, paired *t*-test, *n* = 8, Figures 3A,F). Firing was terminated by DCG-IV in 3 of the 8 cells (Figure 3D). In the 5 cells that continued firing with DCG-IV, firing rate was dramatically reduced from 2.7 ± 0.6 Hz to 0.3 ± 0.1 Hz (*p* < 0.01, paired *t*-test). DCG-IV did not have any discernible effects on spike properties (amplitude, width) and spiking could be restored by depolarizing current injection (*n* = 2; data not shown). Contrasting these effects, the influence of DCG-IV was negligible in 7 mitral cells with apical dendrites that were truncated between the soma and the glomerular layer (Figure 3B). These cells resting membrane potential (−53.7 ± 0.7 mV) was slightly hyperpolarized as compared with intact dendrites (−51.6 ± 0.6 mV, *n* = 17, *p* > 0.11, unpaired *t*-test). DCG-IV did not hyperpolarize (−0.6 ± 0.3 mV, *p* = 0.08, paired *t*-test, Figure 3F) or change the firing frequency (2.8 ± 0.3 Hz vs. 2.8 ± 0.3 Hz, *p* = 0.9, paired *t*-test, Figure 3E) in mitral cells with truncated dendrites. These results suggest that the effects of DCG-IV on mitral cells occur in the glomeruli and require intact apical dendrites.

**Figure 4:**
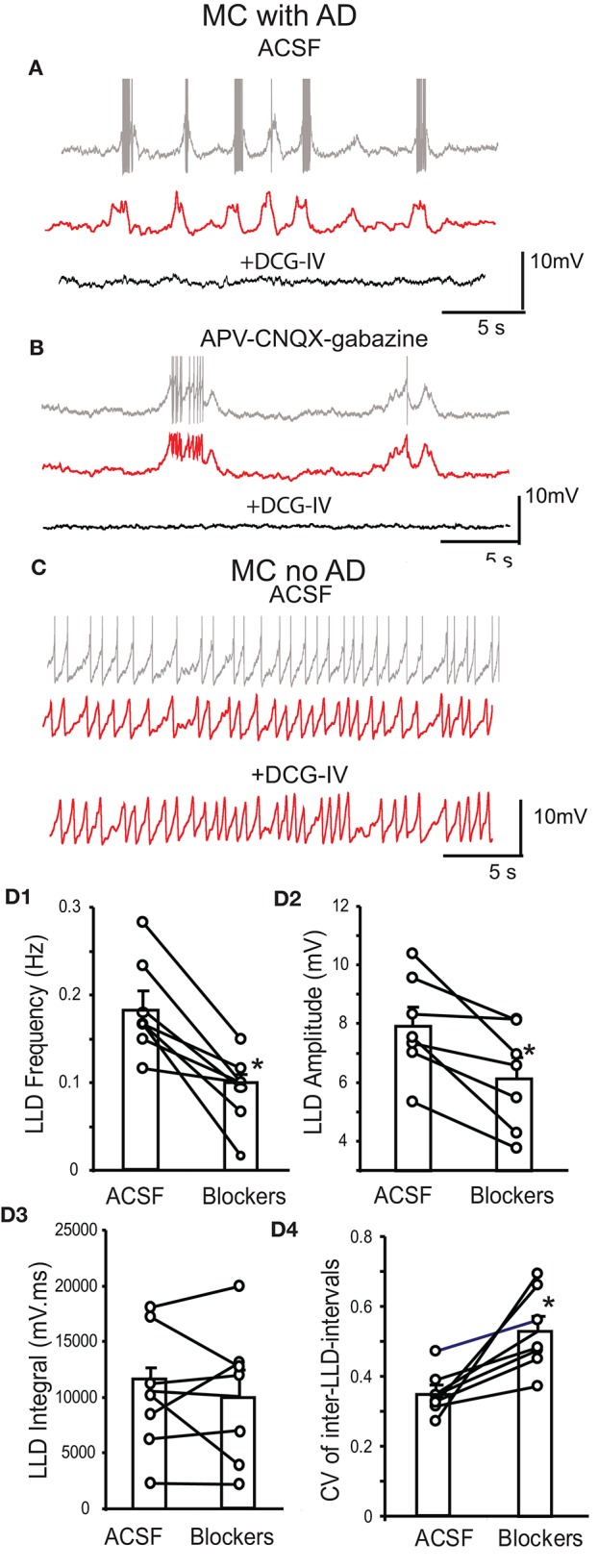
Spontaneous LLDs and membrane potential oscillations in mitral cells. **(A)** Upper trace - example current clamp record shows that a mitral cell (MC) with an intact apical dendrite (AD) in normal ACSF exhibits rhythmic bursting associated with LLDs. Middle trace - low pass filtered record of upper trace more clearly reveals underlying LLDs. Lower trace - DCG-IV eliminates spiking and LLDs. **(B)** In APV-CNQX-gabazine, this mitral cell with intact apical dendrite exhibits spike bursts or single spikes associated with LLD-like membrane potential oscillations (upper trace), as also seen in the middle filtered trace. Lower trace - DCG-IV terminates spiking and membrane potential oscillations. **(C)** An example mitral cell with truncated apical dendrites does not exhibit LLD-like membrane potential oscillations in raw (upper) or filtered traces (middle, lower). **(D1–4)** Mitral cell group data showing LLD properties in normal ACSF and APV-CNQX-gabazine; *n* = 7, ^*^p < 0.05, paired *t*-tests.

DCG-IV produced similar effects in intact mitral cells in the presence of CNQX-APV-gabazine. DCG-IV hyperpolarized mitral cells by −3.4 ± 0.3 mV (*p* < 0.05, *n* = 7 paired *t*-test, Figures 3C,F), a value comparable to that in normal ACSF (*p* = 0.85 unpaired *t*-test, Figure 3F). In all 7 cells, DCG-IV terminated firing (*P* < 0.05, paired *t*-test). Surprisingly, application of CNQX-APV-gabazine alone reduced but did not eliminate mitral cell burst frequency (0.3 ± 0.1 vs. 0.2 ± 0.1 Hz, *p* < 0.05 paired *t*-test, Figure 3B). Since spontaneous LLDs, at least in part, generate spike bursts in mitral cells (De Saint Jan et al., 2009), we wondered what mechanism(s) lead to spike bursts when ionotropic glutamate and GABA receptors are blocked. Close inspection of mitral cell current clamp recordings in the presence of CNQX-APV-gabazine revealed spontaneous depolarizing membrane potential oscillations gave rise to spike bursts or single spikes (Figure 4B). Compared to LLDs in normal ACSF (Figure 4A), these oscillations had a lower amplitude and frequency but a similar integral (Figures 4D1–3). The rise and decay times were somewhat longer, but these trends did not reach statistical significance (rise time: 752.2 ± 172.3 ms vs. 1,012.8 ± 202.3 ms, *p* = 0.13, paired *t*-test; decay time: 1,121.8 ± 328.7 ms vs. 1,179.1±314.7 ms, *p* = 0.89, paired *t*-test). It is possible that small subtle increases in rise and decay time together may have contributed to the stable integral in the two conditions. The oscillations were also less rhythmic than LLDs as reflected by a higher coefficient of variation of inter-LLD intervals (Figure 4D4). Rhythmic LLDs and oscillations were eliminated by DCG-IV (Figures 4A,B), and were not observed in mitral cells lacking intact apical dendrites (Figure 4C).

**Figure 5:**
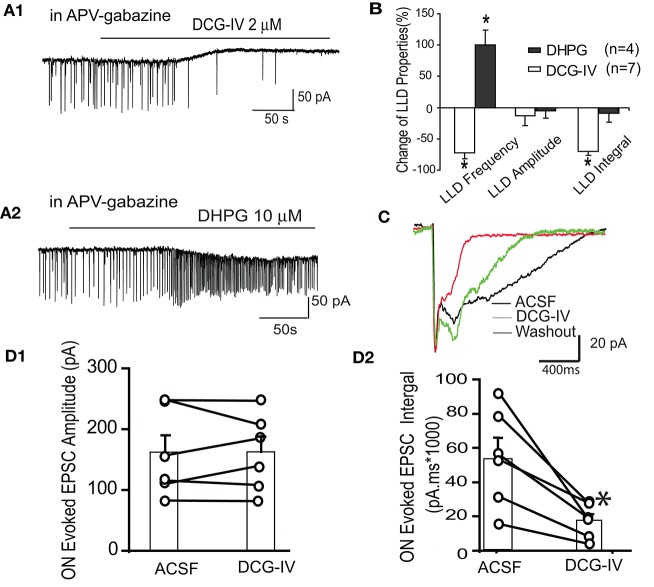
DCG-IV suppresses mitral cell spontaneous and ON-evoked LLDs. **(A)** Example mitral cell voltage clamp records (V_hold_ = −60 mV) in the presence of APV-gabazine show that DCG-IV **(A1)** suppresses while DHPG **(A2)** increases spontaneous LLDs. **(B)** Group data showing the effects of DCG-IV and DHPG on LLD properties. ^*^p < 0.05 vs. respective control. **(C)** In normal ACSF, ON stimulation evoked a rapid monosynaptic EPSC followed by longer duration LLD. The LLD, but not the monosynaptic EPSC, was suppressed by DCG-IV (2 μm). **(D1,2)** Group data (*n* = 6) showing the effects of DCG-IV on the monosynaptic EPSC amplitude **(D1)** and the EPSC integral **(D2)**; ^*^p < 0.05 vs. control.

We further examined the effects of DCG-IV on spontaneous and ON-evoked LLDs in voltage clamp mode. In the presence of APV-gabazine to minimize circuit effects (Figures 5A1,B), DCG-IV induced an outward current at 26.1 ± 8.3 pA, *n* = 6, *p* < 0.05 paired *t*-test) and decreased LLD frequency by 71.6 ± 10.2% (0.29 ± 0.04 Hz to 0.07 ± 0.02 Hz, *p* < 0.01). LLD amplitude was unaffected, but the integral was significantly reduced by 70.2 ± 6.4% (21,965.1 ± 9,131.6 pA.ms vs. 7,721.6 ± 4,288.2 pA.ms, *p* < 0.05, paired *t*-test) resulting from the significant decreases in the kinetics (rise time: 260.7 ± 43.2 ms vs. 120.8 ± 25.2 ms, *p* < 0.05, paired *t*-test; decay time: 453.3 ± 54.2 ms vs. 139.1 ± 20.9 ms, *p* < 0.05, paired *t*-test). We compared the effects of DCG-IV on spontaneous LLDs to the Group I mGluR1 agonist DHPG, which has previously been shown to robustly increase ET cell bursting and mitral cell firing rate (Heinbockel et al., 2004; Dong et al., 2009). The former effect would be expected to increase LLDs in mitral cells. In the presence of APV-gabazine, DHPG increased LLD frequency by ~100% (0.4 ± 0.1 Hz to 0.8 ± 0.1 Hz, *p* < 0.05), but did not alter LLD amplitude or integral (*p* > 0.05; Figures 5A2,B). Thus, DCG-IV and DHPG exert opposing effects on ET and mitral cell spiking in parallel with opposing effects on mitral cell LLDs.

Finally, we tested the effects of DCG-IV on ON-evoked LLDs. In normal ACSF, ON stimuli (30–40 μA) evoked a fast monosynaptic response followed by the delayed LLD as previously reported (Figure 5C; Najac et al., 2011; Shao et al., 2012). DCG-IV (2 μM) did not affect the amplitude (160.2 ± 29.3 pA vs. 161.3 ± 25.6 pA, *p* = 0.92) or rise time (4.6 ± 1.9 ms vs. 6.3 ± 2.3 ms, *p* = 0.29) of the monosynaptic EPSC component (*n* = 6, Figure 5D1). However, EPSC decay time (752.6 ± 231.0 ms vs. 165.0 ± 47.8 ms, *p* < 0.05, paired test) and integral (54,294.9 ± 11,531.0 pA.ms to 17,412.4 ± 4,007.9 pA.ms, *p* < 0.01) were significantly reduced; Figure 5D2). Taken together, these results show that DCG-IV suppresses spontaneous and ON-evoked LLDs in mitral cells, findings consistent with the suppression of ET cell excitability by DCG-IV.

### DCG-IV decreases presynaptic glutamate input to periglomerular (PG) cells

**Figure 6:**
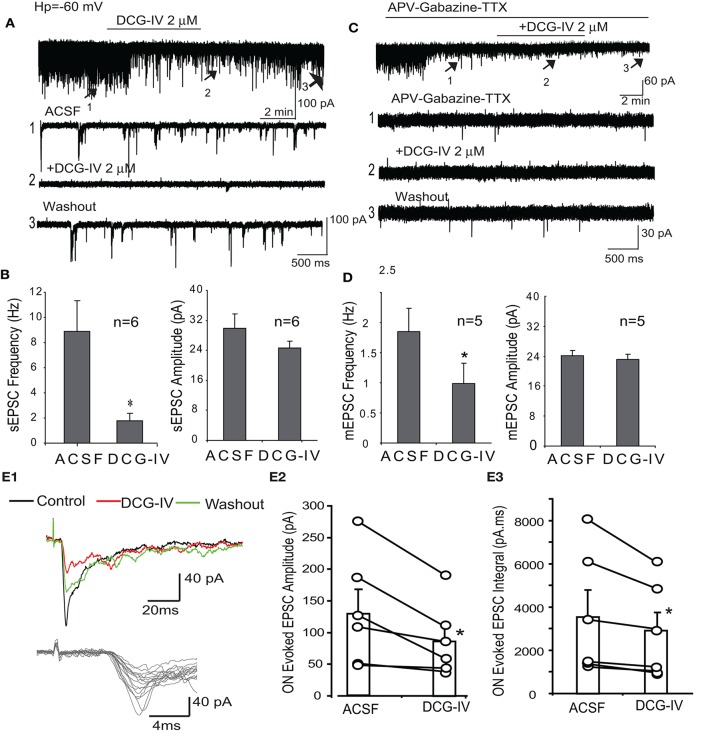
DCG-IV decreases spontaneous and ON-evoked EPSCs in PG cells. **(A)** PG cell voltage clamp recordings (V_hold_ = −60 mV) shows that DCG-IV (2 μM) suppresses sEPSCs. Lower traces show faster time scale records at indicated time points. **(B)** Group data showing that DCG-IV significantly decreases sEPSC frequency but not amplitude; ^*^p < 0.05 compared to control. **(C)** mEPSCs recorded in the presence of APV-gabazine-TTX are suppressed by DCG-IV, as also shown in lower, faster time scale traces. **(D)** Group data showing DCG-IV significantly decreases mEPSC frequency but not amplitude; ^*^p < 0.05, compared to control. **(E1)** ON-evoked polysynaptic EPSCs in a PG cell were suppressed by DCG-IV (2 μM). Lower faster timescale traces show jitter in EPSC onset latency. **(E2,3)** Group data (*n* = 6 PG cells) showing the effect of DCG-IV on EPSC peak amplitude **(E2)** and integral **(E3)**. ^*^p < 0.05 vs. control, paired *t*-test.

The preceding data indicate DCG-IV inhibits ET firing and the delayed (i.e., LLD) but not monosynaptic response of mitral cells to ON input. Suppression of the LLD may be due to reduced ET cell excitability but DCG-IV may also independently suppress dendritic glutamate release from these cells. To distinguish between these two actions, we first recorded spontaneous and miniature EPSCs in periglomerular (PG) cells. Most PG cells (~70%) respond to ON input polsynaptically via from ET cells while ~30% receive monosynaptic input from ON terminals (Hayar et al., 2004b; Shao et al., 2009). These two PG cell classes have distinct electrophysiological signatures: ET-driven PG cells exhibit spontaneous bursts of EPSCs and polysynaptic responses to ON input, whereas ON-driven cells exhibit isolated, single spontaneous EPSCs and monosynaptic ON responses (Shao et al., 2009; Kiyokage et al., 2010). Therefore, EPSC activity in ET-driven PG cells is a sensitive reporter of ET cell glutamate release. Voltage clamp recordings were obtained from PG cells exhibiting characteristic polysynaptic sEPSC bursts (Figure 6A). In normal ACSF (Figures 6A,B), DCG-IV (2 μM, *n* = 6) reduced overall sEPSC frequency by 74.7 ± 8.2% (8.9 ± 2.5 Hz vs. 1.8 ± 0.6 Hz, *p* < 0.05, paired *t*-test,) and sEPSC burst frequency by 85.4 ± 20.0% (0.5 ± 0.2 Hz vs. 0.04 ± 0.21 Hz, *p* < 0.05, paired *t*-test); sEPSC amplitude was not significantly affected (29.9 ± 3.7 pA vs. 24.6 ± 1.9 pA, *p* > 0.05, paired *t*-test, Figure 6B). We next investigated miniature EPCSs (mEPSCs) in the presence of APV (50 μM), gabazine (10 μM) and TTX (1 μM). Under these conditions, bursts of EPSCs were eliminated (*n* = 5, Figure 6C), consistent with the absence of ET cell spiking in TTX (Hayar et al., 2004a; Liu and Shipley, 2008b). mEPSC frequency was also less than that in normal ACSF. DCG-IV significantly decreased mEPSC frequency by 48.6 ± 9.4% (1.9 ± 0.4 Hz vs. 1.0 ± 0.3 Hz (*p* < 0.01, paired *t*-test); mEPSC amplitude (23.8 ± 1.3 pA vs. 22.8 ± 1.5 pA) and kinetics (rise time: 1.6 ± 0.1 ms vs. 1.8 ± 0.1 ms, decay time: 4.3 ± 0.3 ms vs. 4.1 ± 0.3 ms) were not significantly changed (*p* > 0.05, paired *t*-tests, Figure 6D). The reduction of mEPSC frequency, without effects on mEPSC amplitude or kinetics, suggests that DCG-IV presynaptically inhibits ET cell dendritic glutamate release. Finally, DCG-IV did not significantly affect the holding current in these recordings (0.79 ± 1.72 pA, p = 0.67, *n* = 5, paired *t*-test).

We next examined the effects of DCG-IV on PG cell polysynaptic responses to ON input, reasoning that the suppression of ET cell glutamate release would suppress the ON-evoked responses in these ET-driven PGs cells. ON stimulation (30–40 μA) evoked relatively long and variable latency EPSCs in PG cells exhibiting spontaneous EPSC bursts as previously reported (Figure 6E1; Hayar et al., 2004b; Shao et al., 2009). The mean latency of the ON-evoked EPSC was 4.0 ± 0.6 ms, *n* = 6) and the mean jitter (standard deviation of the latency) was 1,322.9 ± 459.2 μs, *n* = 6). DCG-IV (2 μM) significantly decreased EPSCs amplitude (132.9 ± 35.4 pA vs. 88.2 ± 23.3 pA, *p* < 0.05, paired *t*-test) and integral (3,603.7 ± 1,169.3 pA.ms vs. 2,849.2 ± 890.5 pA.ms, *p* < 0.05; *n* = 6, Figure 6E1–3). These results are consistent with presynaptic inhibition of ET cell glutamate release by DCG-IV.

### DCG-IV reduces feedback glutamate release in external tufted cells

**Figure 7:**
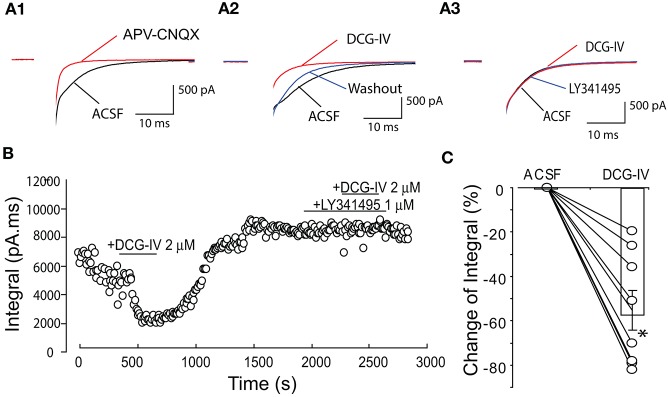
DCG-IV suppressed ET cell self-excitation. **(A1)** Example voltage clamp record showing that a depolarizing step (−60 to 0 mV, 5 ms; artifact blanked) elicited an inward current or self-excitation in this ET cell that was eliminated by APV-CNQX. **(A2)** The evoked current was reversibly suppressed by DCG-IV (2 μM). **(A3)** DCG-IV had no effect in the presence of LY341495 (1 μM). **(B)** Time-running plot showing the integral of the self-excitatory current was reduced by DCG-IV and this effect was blocked by pretreatment of group II mGluR antagonist LY341495. **(C)** Group data from 8 ET cells (open circles) showing the decrease in the normalized inward current integral; bar represents the mean change. ^*^p < 0.05 vs. control, paired *t*-test.

The preceding results indicate that DCG-IV presynaptically reduces glutamate release from ET cells. To further extend these findings, we investigated the effect of DCG-IV on self-excitation elicited by depolarization of single ET cells (−60 to 0 mV, 5 ms duration) in voltage clamp recordings. As shown in Figure 7A1, single depolarizing steps elicited an inward current that was significantly reduced by APV (50 μM) and CNQX (10 μM); 69.8 ± 11.1% reduction of the inward current integral (*n* = 6, *p* < 0.05, paired *t*-test). DCG-IV (2 μM) reversibly reduced the inward current integral (Figures 7A2,B,C) by 55.2 ± 9.0% (4,284.2 ± 1,147.6 pA.ms vs. 1,805.8 ± 463.0 pA.ms, *p* < 0.05, paired *t*-test, *n* = 8). LY341495 (1 μM) itself did not affect the inward current, but prevented the reduction by DCG-IV (ACSF: 4,237.1 ± 2,196.4 pA.ms, LY341495: 4,476.9 ± 2,339.6 pA.ms, LY341495+DCG-IV: 4,219.7 ± 2,208.4 pA.ms; *n* = 3, *p* > 0.19, One way repeated measures ANOVA, Figures 7A3,B). These results provide additional evidence that DCG-IV suppresses glutamate release from ET cells.

## Discussion

The present results indicate that activation of Group II mGluRs directly inhibits ET and mitral cells, and reduces glutamate release from ET cells. These effects occur in the absence of modulation of monosynaptic responses to ON input. However, the inhibition of ET and mitral tufted excitability suppresses intraglomerular glutamate release that generates polysynaptic responses of PG cells to ON input and the generation of LLDs in mitral cells.

DCG-IV uniformly reduced the excitability of ET and mitral cells assessed by membrane potential hyperpolarization and suppression of spontaneous firing. These actions persisted to an equivalent degree in the presence of ionotropic glutamate and GABA receptor antagonists, suggesting a direct effect on the recorded cells. Although Group II mGluRs are well known for presynaptic modulation of neurotransmitter release, they have also been linked to direct membrane hyperpolarization and inhibition of spiking (Bandrowski et al., 2003; Watanabe and Nakanishi, 2003; Govindaiah and Cox, 2006; Mateo and Porter, 2007; Lee and Sherman, 2009; Wang et al., 2012). Inhibition of spiking by DCG-IV was restored by positive current injection, which together with the lack of effect on spike properties, indicate that modulation of fast sodium channels was not involved. The hyperpolarization, and corresponding outward current in voltage clamp, suggest that opening of potassium channels and/or closure of calcium channels near resting threshold may be involved, consistent with cellular actions of Group II mGluRs (Knoflach and Kemp, 1998; Anwyl, 1999; Dutar et al., 2000; Watanabe and Nakanishi, 2003; Lee and Sherman, 2009). Additional studies are needed to establish the ionic basis of Group II mGluRs inhibitory actions on mitral and ET cells. As ET and PG cell processes are confined to the glomerular layer, and DCG-IV effects were absent in mitral cells lacking apical dendrites, the anatomical locus of the Group II mGluR action(s) appear to be in the glomeruli.

In addition to postsynaptic inhibitory actions, several findings indicate that activation of Group II mGluRs presynaptically inhibit glutamate release from ET and possibly mitral cells. First, DCG-IV suppressed spontaneous LLDs in mitral cells and the late “LLD” component of ON-evoked responses. It might be argued that these effects are secondary to inhibition of spiking in ET and mitral cells. However, DCG-IV did not alter the monosynaptic ON response in either cell type, also consistent with lack of presynaptic modulation of ON glutamate release as judged from mEPSC measurements in ET cells. Second, DCG-IV reduced the frequency, but not amplitude or kinetics, of mEPSCs in the class of PG cells that receive indirect ON input via ET cells, i.e., ET-driven PG cells (Hayar et al., 2004b; Shao et al., 2009). DGG-IV also reduced the polysynaptic ON response in these ET-driven PG cells. Third, DCG-IV suppressed evoked glutamate release from individual ET cells. These findings in aggregate are in agreement with presynaptic inhibition of transmitter release by Group II mGluRs in numerous brain circuits (Anwyl, 1999), including glutamate from mitral cells (Schoppa and Westbrook, 1997) and GABA release from granule and PG cells (Zak et al., 2015). Our findings are also consistent with the observations of Zak et al. (2015) that DCG-IV hyperpolarized mitral cells and did not influence ON-evoked monosynaptic EPSCs in ET cells. Our findings however contrast the findings of this study that Group II mGluR activation and inactivation respectively enhanced or inhibited ON-evoked LLDs, and that DCG-IV did not influence PG cell polysynaptic responses to ON stimulation (Zak et al., 2015). The enhancement of ON-evoked LLDs by DCG-IV in the Zak et al. study was attributed to extrasynpatic spillover of glutamate from ET cells that activated Group II receptors on PG cells leading to decreased GABA release, i.e., disinhibition. However, GABAergic disinhibition cannot account for the similar inhibitory effects of DCG-IV observed in normal ACSF, APV-gabazine and APV-CNQX-gabazine in the present study. The differing results of Zak et al. (2015) may be due to the use of younger mice (postnatal day 8–15) and/or the presence of a GABAb receptor antagonist in the ACSF. Additionally, the disinhibitory actions of Group II mGluRs in the Zak et al. (2015) study were in part based on use of an mGluR2 antagonist, which may also potentially underlie differences with the present study.

The prolonged spontaneous depolarizations observed in mitral cells in the presence of APV-CNQX-gabazine have not been previously reported to our knowledge. This depolarization was associated with single spikes or spike bursts. They resemble LLDs observed in normal ACSF with respect to frequency and duration, but are smaller in amplitude and less rhythmic. With a frequency less than 0.3 Hz, they are slower than the 10-20 Hz or gamma frequency subthreshold membrane potential oscillations previously reported for mitral cells *in vitro* (Desmaisons et al., 1999; Friedman and Strowbridge, 2003). They also do not appear to correspond to the upstate of mitral cell bistability, which is typically shorter in duration (20–500 ms) and uniformly terminated by a spike (Heyward et al., 2001). The prolonged depolarizations were absent in mitral cells lacking intact apical dendrites, suggesting a common glomerular origin with LLDs (Carlson et al., 2000). Candidate mechanisms that may generate these depolarization in synaptic blockers are: (1) an excitatory chemical transmitter released in the glomeruli, e.g., excitatory actions of glutamate acting at mGluR1 (Schoppa and Westbrook, 2001; Heinbockel et al., 2004; Yuan and Knopfel, 2006), cholecystokinin (Ma et al., 2013) or β adrenoreceptors (Nai et al., 2010; Zhou et al., 2016); and (2) an intrinsic membrane current such as a sustained sodium current; and/or depolarizations transmitted by electrical synapses between glutamatergic apical dendrites in the glomeruli (Schoppa and Westbrook, 2002; Christie et al., 2005; Hayar et al., 2005; Ma and Lowe, 2007; Pimentel and Margrie, 2008; De Saint Jan et al., 2009).

The suppression of ET and mitral cell excitability by Group II mGluRs in the mature MOB network contrasts with the net excitatory effects of Group I mGluR activation. Group I mGluRs, and in particular mGluR1, contribute to ET and mitral cell excitation in response to ON stimulation *in vitro* (Heinbockel et al., 2004; De Saint Jan and Westbrook, 2005; Ennis et al., 2006; Dong et al., 2009) and odor responses *in vivo* (Matsumoto et al., 2009). These and other studies (Schoppa and Westbrook, 2001; Yuan and Knopfel, 2006) indicate that activation of Group I receptors in the glomerular enhance and amplify ET and mitral/tufted cells responses to ON input and are also recruited by glutamate spillover to enhance the generation of LLDs and slow rhythmic oscillations in the glomerular network. By contrast, the present findings suggest that Group II receptor activation operate in an opposite manner to suppress glutamate spillover in the glomeruli and hence dampen the expression of LLDs and rhythmic oscillations. If Group II mGluRs are preferentially engaged by strong or relatively high frequency input ON input (Zak et al., 2015), perhaps during fast sniffing, this may serve to truncate self- and lateral excitation mediated by intraglomerular glutamate release. This could serve as a gain control to modulate the dynamic range of mitral/tufted cell odor responses, and/or to temporally sharpen spike responses to the sniff rhythm. Together, our data point to another feature of intraglomerular circuits (glutamate spillover, LLDs, electrical synapses, presynaptic inhibition of ON terminals, PG cell-mediated feedback and feedforward inhibition) that function to dynamically modulate the strength and temporal precision of odor responses.

## Author contributions

H-WD designed the study, performed experiments, analyzed the results, wrote the article and approved the final version of the manuscript. ME designed the study, modified the manuscript and approved the final version of the manuscript.

### Conflict of interest statement

The authors declare that the research was conducted in the absence of any commercial or financial relationships that could be construed as a potential conflict of interest.
